# Identification of Novel Host Interactors of Effectors Secreted by *Salmonella* and *Citrobacter*

**DOI:** 10.1128/mSystems.00032-15

**Published:** 2016-07-12

**Authors:** Ryan L. Sontag, Ernesto S. Nakayasu, Roslyn N. Brown, George S. Niemann, Michael A. Sydor, Octavio Sanchez, Charles Ansong, Shao-Yeh Lu, Hyungwon Choi, Dylan Valleau, Karl K. Weitz, Alexei Savchenko, Eric D. Cambronne, Joshua N. Adkins

**Affiliations:** a Biological Sciences Division, Pacific Northwest National Laboratory, Richland, Washington, USA; b Department of Molecular Microbiology and Immunology, Oregon Health and Science University, Portland, Oregon, USA; c Department of Veterinary Microbiology and Pathology, Washington State University, Pullman, Washington, USA; d Saw Swee Hock School of Public Health, National University of Singapore, Singapore; e Department of Chemical Engineering and Applied Chemistry, Banting, and Best Department of Medical Research, Midwest Centre for Structural Genomics, University of Toronto, Toronto, Ontario, Canada; University of Hawaii

**Keywords:** affinity purification, mass spectrometry, effectors, pathogenic bacteria, type III secretion system, protein-protein interactions

## Abstract

Many pathogenic bacteria of the family *Enterobacteriaceae* use type III secretion systems to inject virulence proteins, termed “effectors,” into the host cell cytosol. Although host-cellular activities of several effectors have been demonstrated, the function and host-targeted pathways of most of the effectors identified to date are largely undetermined. To gain insight into host proteins targeted by bacterial effectors, we performed coaffinity purification of host proteins from cell lysates using recombinant effectors from the *Enterobacteriaceae* intracellular pathogens *Salmonella enterica* serovar Typhimurium and *Citrobacter rodentium*. We identified 54 high-confidence host interactors for the *Salmonella* effectors GogA, GtgA, GtgE, SpvC, SrfH, SseL, SspH1, and SssB collectively and 21 interactors for the *Citrobacter* effectors EspT, NleA, NleG1, and NleK. We biochemically validated the interaction between the SrfH *Salmonella* protein and the extracellular signal-regulated kinase 2 (ERK2) host protein kinase, which revealed a role for this effector in regulating phosphorylation levels of this enzyme, which plays a central role in signal transduction.

**IMPORTANCE** During infection, pathogenic bacteria face an adverse environment of factors driven by both cellular and humoral defense mechanisms. To help evade the immune response and ultimately proliferate inside the host, many bacteria evolved specialized secretion systems to deliver effector proteins directly into host cells. Translocated effector proteins function to subvert host defense mechanisms. Numerous pathogenic bacteria use a specialized secretion system called type III secretion to deliver effectors into the host cell cytosol. Here, we identified 75 new host targets of *Salmonella* and *Citrobacter* effectors, which will help elucidate their mechanisms of action.

## Abstract (precis)

During infection, pathogenic bacteria face an adverse environment of factors driven by both cellular and humoral defense mechanisms. To help evade the immune response and ultimately proliferate inside the host, many bacteria evolved specialized secretion systems to deliver effector proteins directly into host cells. Translocated effector proteins function to subvert host defense mechanisms. Numerous pathogenic bacteria use a specialized secretion system called type III secretion to deliver effectors into the host cell cytosol. Here, we identified 75 new host targets of *Salmonella* and *Citrobacter* effectors, which will help elucidate their mechanisms of action.

## INTRODUCTION

Many pathogenic bacteria have evolved specialized virulence proteins, known as effectors, which are secreted into the host during the infection process. These proteins modulate a variety of host-driven processes for the benefit of the bacterium, such as immune evasion or modulation of the host cell to promote a replication-permissive environment. Effectors drive many of the physiological effects that are attributed to an infection. Thus, it is desirable to elucidate the interactions occurring between the effector protein and its host target(s) (1 – 3). Translocation of effectors from the bacterium into the host is achieved via a variety of mechanisms, including specialized secretion systems, the flagellum, and vesicular secretion (4).

The type III secretion system (T3SS) evolved from the flagellar apparatus and represents a common mechanism for secretion of effector proteins. Consequently, it is used by many Gram-negative bacteria to support pathogenesis (5, 6). The T3SSs are particularly common among the pathogenic members of the *Enterobacteriaceae* family of bacteria, which includes the genera *Salmonella*, *Escherichia*, *Yersinia*, *Klebsiella*, *Enterobacter*, and *Citrobacter*. Interestingly, effectors usually have low sequence similarity to other proteins; even orthologous effectors of related pathogens have limited sequence identity (7, 8). This phenomenon is partly driven by horizontal gene transfer events with apparent strong selective pressures for effectors mediated by host defense processes rather than inheritance from a common ancestor (9). While bacterial effectors and their host interaction partners have been studied for some time, the diversity of effectors offers opportunities to systematically discover and evaluate interactions between effectors and host proteins. Protein-protein interaction screens, including yeast two-hybrid (Y2H) assays, fluorescent resonance energy transfer (FRET) analyses, mutagenesis with phenotype assays, and coimmunoprecipitations (co-IP), have increased our understanding of effector-driven pathogenesis (10). An alternative technique is that of affinity purification (AP) followed by mass spectrometry (AP-MS) (11), which is highly sensitive and benefits greatly from parallelization to provide greater specificity of interactions (12). In addition, the use of known contaminant protein databases (13) and of statistical methods (14) has further improved the confidence of identifications and reduced the false-discovery rates of AP-MS.

Here we selected a set of effectors from two *Enterobacteriaceae* species, *Salmonella enterica* serovar Typhimurium and *Citrobacter rodentium*, to apply an AP-MS method, leading to the identification of 75 effector-host protein interactions. We validated several effector-host protein interactions, including a previously characterized interaction, such as the *Salmonella* effector SspH1—host kinase PKN1 interaction, by Western blot analysis. To illustrate the potential of our approach, we further studied the interaction between the host mitogen-activated protein (MAP) kinase, extracellular signal-regulated kinase 2 (ERK2), and *Salmonella* secreted effector protein SsrB regulated factor H (SrfH), also known as *Salmonella* secreted effector I (SseI). This interaction was shown to also affect the phosphorylation of ERK2, which implies a role in the regulation of this kinase’s activity.

## RESULTS AND DISCUSSION

### Identification of novel host binding partners of bacterial secreted effectors by AP-MS.

**FIG 1:**
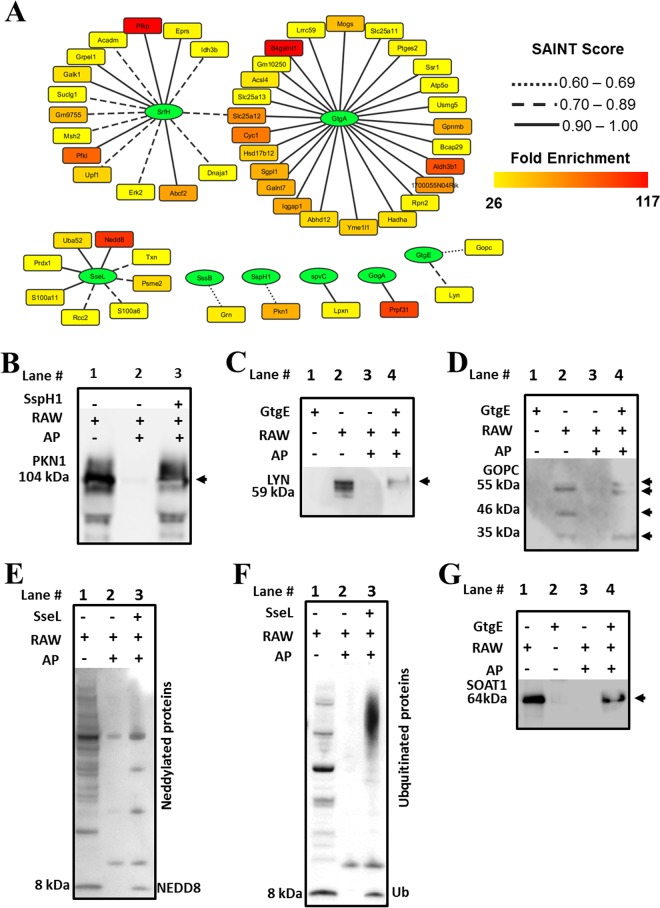
Identification of host targets of *Salmonella* secreted effector proteins. (A) Network of *Salmonella* secreted effector proteins (green elliptical nodes) and host targets (rectangle nodes). The host targets are colored according to their fold enrichment in the affinity purification compared to control levels, and the edges are represented according to the significance score calculated by SAINT. (B to G) Several identified interacting pairs were validated by Western blot experiments as follows. (B) *Salmonella* effector SspH1 and host kinase PKN1. (C) *Salmonella* effector GtgE and host tyrosine kinase LYN. (D) *Salmonella* effector GtgE and host sterol *O*-acyltransferase (SOAT1). (E) *Salmonella* secreted deubiquitinase SseL and ubiquitin-like protein NEDD8. (F) *Salmonella* secreted deubiquitinase SseL and ubiquitin. (G) *Salmonella* effector GtgE and Golgi-associated PDZ and coiled-coil motif-containing (GOPC) protein. Abbreviations: AP, affinity purification; RAW, cell lysate of RAW 264.7 cells.

To identify host targets of bacterial effectors, we used recombinant proteins from two members of the *Enterobacteriaceae* family, *Citrobacter rodentium* and *Salmonella* Typhimurium, as bait for *in vitro* AP-MS. Since *C. rodentium* infects epithelial cells and since *S*. Typhimurium infects leukocytes and epithelial cells, APs were performed using HeLa and RAW 264.7 macrophage-like cell lysates, respectively. Following AP, samples were digested with trypsin and subjected to liquid chromatography-tandem mass spectrometry (LC-MS/MS) for peptide identification and quantification. Then, we filtered the resulting data using the abundance of interacting proteins in the AP sample relative to that of the no-bait control and the statistical score provided by the SAINT (significance analysis of the interactome) tool (15). The well-characterized interaction between the *Salmonella* effector SspH1 and the host protein kinase PKN1 (16, 17) was used as a guide to filter the data that included proteins with at least 10-fold enrichment in the affinity purification versus non-bait-containing samples and a SAINT probability score larger than 0.6 (Fig. 1; see also Table S1 in the supplemental material). Data from host proteins that interacted with more than 3 effectors (12) or had above 10 spectral counts on average in the CRAPome database (13) were considered to represent nonspecific interactions and were also filtered out of the final data set. Since many of the interactions that failed to meet these criteria can represent false negatives, they were separated into a group of intermediate-confidence interactions. Interactors that were highly (>10-fold) enriched in the affinity purification but had a poor SAINT probability score and interactors that had a high (>0.6) SAINT score but showed lower (<10-fold) enrichment were considered to represent intermediate-confidence interactions (see Table S1). The intermediate-confidence interactors also included hits that were filtered out on the basis of their binding to multiple bait proteins and of their presence in the CRAPome database.

10.1128/mSystems.00032-15.5 Table S1  Identified *Salmonella* effector-host protein interactions. *Salmonella* effectors fused to SBP tags were submitted to coaffinity purifications using RAW 264.7 cell lysates and analyzed by liquid chromatography-tandem mass spectrometry. Quantitative analysis was performed with spectral counts, and interactions were tested for significance using SAINT. Download Table S1, XLSX file, 0.03 MB. Copyright © 2016 Sontag et al. 2016 Sontag et al. This content is distributed under the terms of the Creative Commons Attribution 4.0 International license.

### Host proteins targeted by *Salmonella* effectors.

*Salmonella* effectors GogA, GtgA, GtgE, SpvC, CigR, PipB2, SifA, SrfH, SseL, SspH1, SssA, and SssB, which represent several well-characterized (18 – 25) and recently identified (26) bacterial effectors, were used as bait proteins (Fig. 1; see also Table S1 in the supplemental material). A total of 54 protein-protein interactions with the GogA, GtgA, GtgE, SpvC, SrfH, SseL, SspH1, and SssB effectors were identified, whereas no significant interactor was detected for CigR, PipB2, SifA, and SssA (Fig. 1A). It is possible that some of these effectors need cofactors or processing not available in an *in vitro* system. We next validated several of the effector-host protein interactor pairs by performing AP followed by Western blot analyses, with the known SspH1 effector and PKN1 host protein interactor pair included as a positive control (Fig. 1B). We also verified an interaction between GtgE and host LYN tyrosine kinase and multiple isoforms of Golgi-associated PDZ and coiled-coil motif-containing (GOPC) protein (Fig. 1C and D). Since GtgE was shown to be a secreted protease that targets host Rab29 and Rab32 (27, 28), we tested whether this effector would cleave the GOPC protein. However, we found no evidence of GOPC cleavage by GtgE, at least in *in vitro* experiments performed using recombinant proteins and cell lysates (data not shown).

Since our group recently identified novel substrates for *Salmonella* secreted deubiquitinase SseL (29), S100A6, and heterogeneous nuclear ribonucleoprotein M (hnRNP M), we further interrogated the interacting partners via AP-MS. We were able to confirm an SseL-S100A6 interaction in this new data set but not an hnRNP K interaction. This could have been due to the fact that only a small portion of ubiquitinated hnRNP K specifically binds to SseL, whereas some unmodified forms bind nonspecifically to the beads to some extent (29). Another interesting putative interactor was NEDD8, a ubiquitin-like protein modifier, which was also confirmed by Western blot analysis (Fig. 1E). We further investigated whether or not SseL has deNEDDylase activity by incubating cell lysates with recombinant SseL followed by Western blot analysis. No deNEDDylase activity was detected (see Fig. S1 in the supplemental material); however, there is a possibility that SseL is very specific to a small number of NEDDylated substrates. Interestingly, SseL seems to interact with free (unconjugated) NEDD8. Considering that NEDD8 is a homolog of ubiquitin, a reaction product of SseL activity, and considering that reaction products should be released by the enzyme, it is reasonable to think that SseL should not interact with free ubiquitin or NEDD8. Therefore, we also tested the ability of SseL to interact with free ubiquitin. This interaction was validated by Western blotting (Fig. 1F). Since it is unusual for enzymes to interact with reaction products, we speculate that SseL may harbor activities involved in modulating the ubiquitination pathway in addition to the previously observed deubiquitination activity (24).

10.1128/mSystems.00032-15.1 Figure S1  DeNEDDylase enzyme assay with *Salmonella* deubiquitinase SseL. RAW 264.7 cell lysate was incubated for 15 min with 2 µg SseL/mg lysate for 15 min (A) or 20 µg SseL/mg lysate for 18 h (B) and analyzed by Western blotting using anti-NEDD8 antibody. M, molecular weight marker; 1, untreated control; 2, lysate treated with SseL. Download Figure S1, TIF file, 0.3 MB. Copyright © 2016 Sontag et al. 2016 Sontag et al. This content is distributed under the terms of the Creative Commons Attribution 4.0 International license.

In the pool of intermediate-confidence interactors, 227 interacting pairs were identified for all of the tested effectors, with the exception of SssA (see Table S1). This pool of proteins contained hits such as tubulin, which is considered a common contaminant in AP-MS experiments (13), and heat shock proteins that are known to bind to unfolded proteins (30). Despite the low scores, several of the interacting pairs from this pool might represent true positives, although it is virtually impossible to distinguish them from the false positives just by statistical analysis. To illustrate the utility of this pool of intermediate-confidence interactions, we performed a Western blot analysis and were able to validate the interaction between GtgE and sterol *O*-acyltransferase (SOAT1) (Fig. 1G).

### Host proteins targeted by *Citrobacter* effectors.

**FIG 2:**
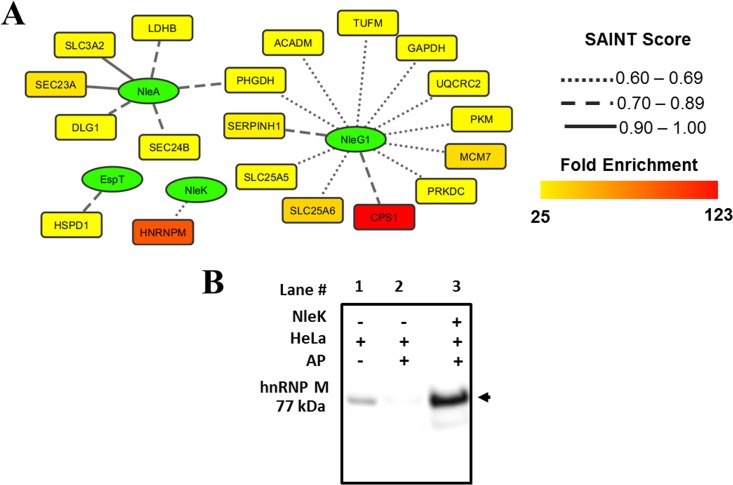
Identification of host targets of *Citrobacter* secreted effector proteins. (A) Network of *Citrobacter* secreted effector proteins (green elliptical nodes) and host targets (rectangle nodes). The host targets are colored according to their fold enrichment in the affinity purification compared to control levels, and the edges are represented according to the significance score calculated by SAINT. (B) Validation of the interaction between the *Citrobacter* effector NleK and the host factor hnRNP M by affinity purification and Western blotting.

*Citrobacter* effectors EspA, EspT, NleA, NleC, NleE, NleG1, NleG8, and NleK were used as bait proteins in AP experiments using HeLa cell lysates. Using the same filters as those applied to the *Salmonella* data, a total of 21 interacting proteins corresponding to EspT, NleA, NleG1, and NleK were identified (Fig. 2; see also Table S2 in the supplemental material). In addition, 157 interacting proteins against all tested *Citrobacter* effectors were identified with intermediate confidence (see Table S2). Among the identified high-confidence interacting partners, we were able to verify the previously described interaction between *Citrobacter* NleA and the protein transport protein Sec23A (31) (Fig. 2A). Among the newly identified interactions, we validated the interaction between NleK and heterogeneous nuclear ribonucleoprotein M (hnRNP M) by AP and subsequent Western blotting (Fig. 2B). Interestingly, hnRNP M protein is known to be targeted by viral proteases facilitating infection (32), but its function during bacterial infections is largely unknown.

10.1128/mSystems.00032-15.6 Table S2  Identified *Citrobacter* effector-host protein interactions. *Citrobacter* effectors fused to SBP tags were submitted to coaffinity purifications using HeLa cell lysates and analyzed by liquid chromatography-tandem mass spectrometry. Quantitative analysis was performed with spectral counts, and interactions were tested for significance using SAINT. Download Table S2, XLSX file, 0.02 MB. Copyright © 2016 Sontag et al. 2016 Sontag et al. This content is distributed under the terms of the Creative Commons Attribution 4.0 International license.

### *Salmonella* effector SrfH interaction with host kinase ERK2.

**FIG 3:**
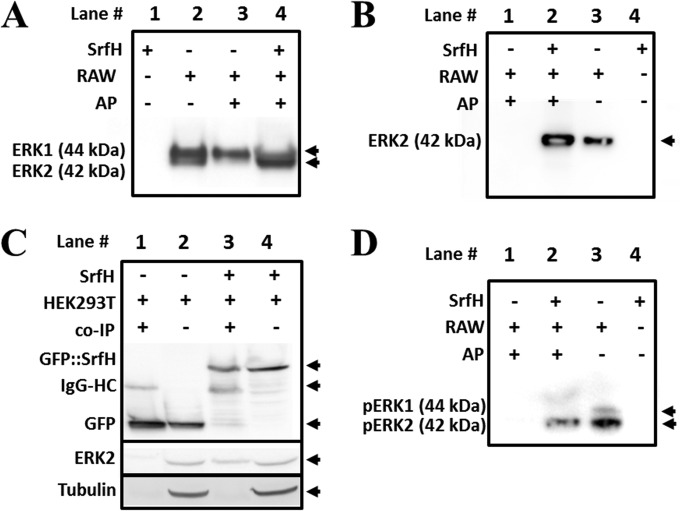
Validation of *Salmonella* effector SrfH interaction with the host kinase ERK2. (A and B) Affinity purification of RAW 264.7 cell lysates using SrfH as bait and analysis by Western blotting using anti-ERK antibodies (A) or anti-ERK2 antibodies (B). (C) Coimmunoprecipitation (co-IP) of HEK293T cells transfected with green fluorescent protein (GFP) fused to SrfH (GFP::SrfH) or GFP alone and probed against GFP, ERK2, IQGAP, and tubulin. (D) Affinity purification of RAW 264.7 cell lysates using SrfH as bait and analysis by Western blotting using anti-phospho-ERK antibodies.

SrfH showed a significant interaction with ERK2 kinase by AP-MS. ERK2 (extracellular signal-regulated kinase 2) is a mitogen-activated protein (MAP) kinase that plays a central roles in eukaryotic cell cycle, cell migration, and phagocytosis (33). Furthermore, previous work has shown that SrfH is an important effector for systemic infection of *Salmonella* Typhimurium (34), which motivated us to further study this interaction. Since ERK1 and ERK2 are highly conserved, it is possible that SrfH could bind to both. We performed an immunoprecipitation experiment using SrfH as bait against RAW 264.7 cell lysate and probed the interaction by Western blotting using an antibody that recognizes both ERK1 (44 kDa) and ERK2 (42 kDa). The results showed an enrichment of the band at 42 kDa, while some nonspecific binding of ERK1 (44 kDa) was also observed (Fig. 3A), suggesting that SrfH has a preference for ERK2. We also performed a Western blot experiment, using a monoclonal antibody specific to the ERK2 isoform, which demonstrated the enrichment of the 42-kDa band (Fig. 3B). To further confirm this interaction, we expressed green fluorescent protein (GFP) alone or fused to SrfH (GFP::SrfH) in HEK293T cells and performed coimmunoprecipitation (co-IP) using anti-GFP antibodies. The results demonstrated that ERK2 was enriched in AP with GFP::SrfH but not GFP alone (Fig. 3C). We next examined if SrfH could bind active (phosphorylated) forms of ERK1 and ERK2 by performing AP of SrfH against RAW 264.7 cell lysates and a Western blot experiment using an antibody that recognizes the phosphorylated forms of both ERK2 and ERK1. As observed for the total ERK forms, SrfH interacted with the phosphorylated form of ERK2 but not with that of ERK1 (Fig. 3D). Collectively, these results demonstrated that SrfH bound to both the nonphosphorylated and phosphorylated forms of ERK2 but not to those of the paralogous ERK1.

**FIG 4:**
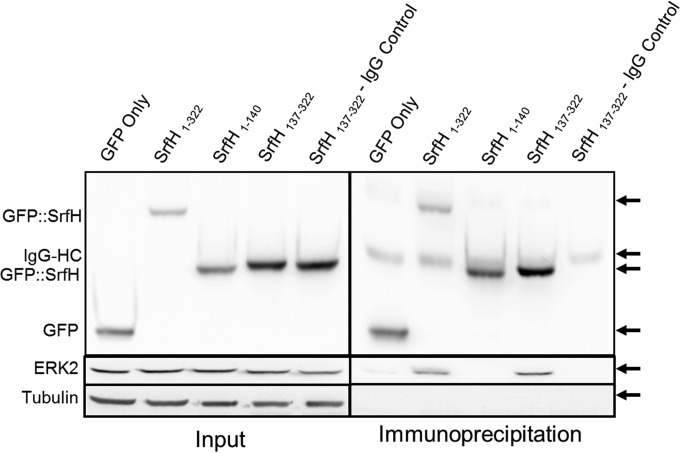
ERK2 interactions with different SrfH domains. HEK293T cells transfected with green fluorescent protein (GFP) fused to full-length or truncated forms of SrfH (GFP::SrfH) or GFP alone were coimmunoprecipitated (co-IP) with anti-GFP antibodies and probed against GFP, ERK2, and tubulin.

Having validated the interaction, we sought to define the region of SrfH that bound to ERK2. HEK293 cells were transfected with a plasmid encoding GFP alone (GFP) or fused to the N terminus (GFP::SrfH 1 to 140) or C terminus (GFP::SrfH 137 to 322) of SrfH. Co-IP and Western blot experiments were performed using an anti-GFP antibody. Enrichment of ERK2 was detected only when full-length SrfH or the C-terminal construct of SrfH was present, indicating that the binding domain occurs within the C-terminal 185-amino-acid region of SrfH (Fig. 4).

SrfH has three well-characterized host targets: thyroid receptor interacting protein 6 (TRIP6), IQ containing GTPase activating protein 1 (IQGAP1), and filamin (34 – 36). Interestingly, binding to these targets has been proposed to have very different roles during infection, although they are all related to cytoskeleton and cellular movement. Whereas SrfH binding to TRIP6 has been reported to promote phagocyte mobility and systemic dissemination, IQGAP1 interactions were shown to suppress *Salmonella* migration to the spleen (34, 35). These contradictory phenotypes might be due to a single nucleotide polymorphism that dictates SrfH binding to TRIP6 (37). SrfH from *Salmonella* Typhimurium 14028s bears a glycine residue at position 103, whereas the allele from serovar SL1344 has an aspartic acid residue at the same position which abolishes the binding to TRIP6 (37). The relationships among different SrfH targets are still unknown, but, interestingly, IQGAP1 is known to be regulated by ERK2 (38) whereas TRIP6 is regulated by other kinases (39). Thus, it is possible that IQGAP1 and TRIP6 are regulated directly or indirectly by SrfH action on ERK2.

### SrfH regulates ERK2 phosphorylation.

**FIG 5:**
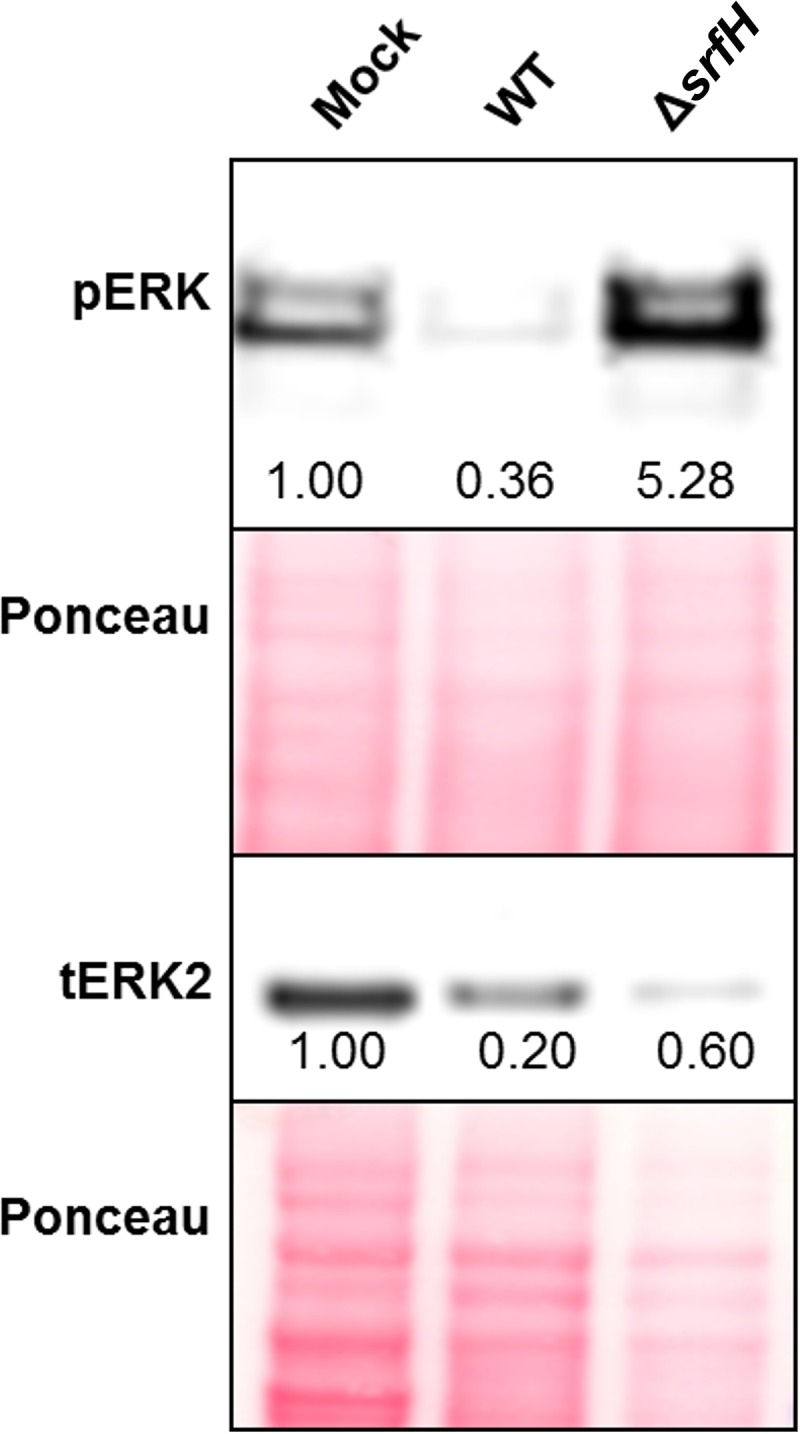
Regulation of ERK2 phosphorylation levels by SrfH. RAW 264.7 cells were infected with either the wild-type (WT) strain or a SrfH deletion strain (Δ*srfH*), lysed, and analyzed by Western blotting using anti-phospho-ERK (pERK) or anti-total ERK2 (tERK2) antibodies. A relative densitometry value, normalized to that of the loading (Ponceau stain), is listed below each band.

To gain further insight into the interaction between SrfH and ERK2, we aimed to determine if SrfH affected the levels of ERK2 phosphorylation during infection. RAW 264.7 cells were infected with either wild-type (WT) or Δ*srfH* mutant *S*. Typhimurium for 24 h, after which cells were harvested and lysed in the presence of phosphatase inhibitors. The lysates were analyzed by Western blotting to determine if total ERK or phospho-ERK levels were altered in either proteoform. While the levels of total ERK2 were slightly reduced (relative to that of total protein), levels of phospho-ERK were dramatically decreased in the infection with the wild-type strain compared to the mutant infection results (Fig. 5). These results suggested that SrfH decreases the level of phospho-ERK or somehow prevents ERK from being phosphorylated in RAW 264.7 cells. Interestingly, the levels of phosphorylated ERK2 and ERK1 were similarly altered, although we did not detect a direct interaction between SrfH and ERK1 (Fig. 3). We speculate that regulation of ERK1 by SrfH could be an indirect effect. It is also worth noting that the phospho-ERK antibodies used in our study target sites that activate the enzyme when phosphorylated; thus, the reduction of phosphorylation levels by SrfH suggests an inhibition of ERK2 activity.

Structural elucidation of SrfH showed similarities to enzymes containing cysteine in the active site, including cysteine protease, glutamine deamidase, and transglutaminase (40). Thus, to try to find a potential mechanism of action, we tested the possibility that SrfH is a protease or glutamine deamidase but found no supporting evidence (see Text S1 and Fig. S2 to S3 in the supplemental material). Since SrfH decreased the levels of ERK2 phosphorylation in RAW 264.7 cells infected with *Salmonella*, we examined whether SrfH had phosphatase or irreversible phosphorylase activity, as previously shown for *Salmonella* secreted effectors SptP and SpvC, known to interact with ERK2 (41, 42). Thus, we examined the levels of phosphorylated peptides of ERK2 after treatment with SrfH. Although a total of three phosphopeptides, corresponding to three modification sites, were identified and quantified, none of them decreased in abundance after treatment with SrfH (see Fig. S4), suggesting that SrfH did not act directly on ERK2 phosphorylation sites. We cannot exclude the possibility that host cofactors not present *in vitro* assays may be required for enzymatic activity. Interestingly, SpvC, SptP, and SrfH target ERK2. This targeting may appear redundant, but these effectors have distinct localizations within the host cell. While SrfH targets the plasma membrane (43), SpvC is evenly distributed in the cytoplasm (42), and SptP resides within the *Salmonella*-containing vacuoles (44), suggesting that these effectors modulate different ERK2-driven pathways. Indeed, whereas SpvC decreases inflammation in the host gut, SrfH has a role in the pathogen dissemination (34, 35, 45).

10.1128/mSystems.00032-15.2 Figure S2  *In vitro* proteolysis assay. Recombinant ERK2 was incubated with different amounts of SrfH in the presence or absence of different protease inhibitors, namely, metalloprotease inhibitor EDTA, proteinase inhibitor cocktail (HALT), and cysteine protease inhibitor N-ethylmaleimide (NEM). Reaction products were analyzed by Western blotting using anti-ERK2 antibodies. *, the molecular weight shift seen with NEM-treated samples might have been due to alkylation of ERK2 cysteine residues by this reagent. Download Figure S2, TIF file, 0.1 MB. Copyright © 2016 Sontag et al. 2016 Sontag et al. This content is distributed under the terms of the Creative Commons Attribution 4.0 International license.

10.1128/mSystems.00032-15.3 Figure S3  Quantification of glutamine and asparagine deamidation sites on ERK2. ERK2 was incubated in different ratios with SrfH, and products were separated by SDS-PAGE and analyzed by LC-MS/MS. Peptide identification and extraction of peak areas were performed with MaxQuant. The abundance of each deamidated peptide was normalized to that of its unmodified version. No significant changes were observed by Student’s *t* test. The data in this figure represent averages of the results of two independent experiments performed in technical replicates. Download Figure S3, TIF file, 0.3 MB. Copyright © 2016 Sontag et al. 2016 Sontag et al. This content is distributed under the terms of the Creative Commons Attribution 4.0 International license.

10.1128/mSystems.00032-15.4 Figure S4  Quantification of phosphorylation sites on ERK2. ERK2 was incubated in different ratios with SrfH, and products were separated by SDS-PAGE and analyzed by LC-MS/MS. Peptide identification and extraction of peak areas were performed with MaxQuant. The abundance of each phosphorylated peptide was normalized to that of its unmodified version. No significant decrease on phosphorylation levels were observed by Student’s *t* test. The data in this figure represent averages of the results of two independent experiments performed in technical replicates. Download Figure S4, TIF file, 0.1 MB. Copyright © 2016 Sontag et al. 2016 Sontag et al. This content is distributed under the terms of the Creative Commons Attribution 4.0 International license.

10.1128/mSystems.00032-15.7 Text S1  Supplemental Materials and Methods, Results, and Discussion. Download Text S1, DOCX file, 0.03 MB. Copyright © 2016 Sontag et al. 2016 Sontag et al. This content is distributed under the terms of the Creative Commons Attribution 4.0 International license.

### Conclusions.

Screening approaches for protein-protein interactions have different strengths and weaknesses. It is possible to identify interactions using a variety of methodologies, including yeast two-hybrid assays, protein microarrays (46), and AP-MS (11). Validation of an interaction generated using one method can often be difficult to confirm using a second assay. Affinity tags, antibodies, and/or constructs can often induce or abrogate specific interactions. However, these tools can be incredibly powerful as the foundation for new studies of mechanism function. Here we utilized AP-MS to identify, validate, and provide more-detailed insights into bacterial secreted effector proteins and their host binding targets. This approach was highlighted by our investigation of the SrfH effector protein, where we examined potential enzymatic activities and functional consequences of interactions with ERK2. In total, we confidently identified 75 secreted effector protein-host protein interactions. These newly identified host-interacting partners can serve as a resource and will hopefully help clarify how these pathogenic bacteria subvert specific aspects of the host-cellular environment.

## MATERIALS AND METHODS

### Cloning, expression, and purification of SBP-tagged proteins.

Effectors were amplified on the basis of sequences of *Salmonella* Typhimurium strain 14028s and *Citrobacter rodentium* genomic DNA. Then, each gene was cloned in pET vectors containing His6/streptavidin-binding peptide (SBP) and a tobacco etch virus (TEV) protease cleavage site fused to its N terminus and was expressed in *Escherichia coli* strain BL21. Expression and purification of each bait protein were performed as described previously in detail (29).

### Cell culture/lysate preparation.

RAW 264.7 macrophage-like cells (ATCC tib-71; ATCC, Manassas, VA) were cultured in Dulbecco’s modification of Eagle’s medium, whereas HeLa cells (ATCC ccl-2) were cultured in minimal essential medium; both cell lines were supplemented with 10% fetal bovine serum, 100 IU/ml penicillin, and 100 µg/ml streptomycin. Cells were maintained in an atmosphere of 95% humidity and 5% CO_2_ at 37°C before passage 15. Cells were harvested in a reaction mixture consisting of phosphate-buffered saline (PBS) containing 1% Triton X-100, 1× HALT protease inhibitor (Thermo Scientific, Rockford, IL), 5 mM EDTA, and 20 mM sodium orthovanadate or of 50 mM HEPES containing 1% Triton X-100, 1× HALT protease inhibitor, 5 mM EDTA, 5 mM *N*-ethylmaleimide (NEM), and 0.5 mM Tris(2-carboxyethyl)phosphine (TCEP) for SseL pulldown. Proteins were extracted by sonication at 100% amplitude using pulses of on/off cycles of 0.5 s each for 3 repetitions at 30 s per repetition along with vigorous vortex mixing. The crude lysate was clarified by centrifugation at 10,000 × *g* for 15 min at 4°C, snap frozen in liquid N_2_, and stored at −80°C.

### Affinity purification, protein digestion, and Western blot analysis.

RAW 264.7 or HeLa whole-cell lysate (7.5 mg) was combined with 4 nM purified, SBP-tagged effector protein in a streptavidin-binding buffer (SBB) containing 5 mM 2-mecaptoethanol, 2 mM EDTA, and 0.1% (vol/vol) Triton X-100 at pH 7.4. This solution was incubated at 4°C, overnight, with end-over-end rotation to allow the effectors and host targets to interact. Then, effector-host protein complexes were captured by adding 50 µl of High Capacity streptavidin agarose resin (Thermo Scientific, Rockford, IL) and rotating at 4°C for 1 h. The beads were harvested by centrifugation at 2,500 × *g* for 2 min and washed twice with SBB to remove noninteracting proteins.

For LC-MS/MS analysis, the washing step was repeated twice with PBS before elution of the bound proteins with 300 µl of elution buffer (2 nM biotin–SBB) was performed for 1 h with rotation. Eluted protein complexes were precipitated with 1 ml of ice-cold acetone overnight. After they were dried to completeness in a SpeedVac concentrator, samples were suspended in 50 µl of a mixture of 100 mM NH_4_HCO_3_, 8 M urea, and 1 mM TCEP and were incubated at 55°C with shaking for 15 min. Free thiols were alkylated by addition of 5 µl of 100 nM NEM and were incubated at 37°C with shaking for 1 h, after which samples were diluted 8-fold with 25 mM NH_4_HCO_3_ to decrease the urea concentration to 1 M. Trypsin (0.5 µg) and CaCl_2_ (1 nM final concentration) were added, and digestion was allowed to proceed overnight at 37°C. Peptides were desalted by C18 solid-phase extraction using Bond Elut Omix tips (Agilent), dried, and suspended in 20 µl of 0.1% formic acid.

For Western analysis, independent experiments were performed similarly to the AP-MS ones, with the difference that the beads were washed once with PBS after the SBB washes and boiled for 10 min in lithium dodecyl sulfate (LDS) sample buffer containing 5 mM TCEP to elute the proteins. Proteins were then separated on 4% to 12% bis-Tris polyacrylamide gels and transferred to a polyvinylidene difluoride (PVDF) membrane for immunoblot analysis. Membranes were blocked and antibodies diluted with 5% (wt/vol) nonfat dry milk–PBS–0.1% Triton X-100. Primary and secondary antibodies were diluted using a dilution range of 1:200 to 1:3,000, according to the manufacturer’s recommendations. Images were acquired on a FluorChemQ system (Bio-Techne, Minneapolis, MN). Due to the propensity of NleK to bind to the beads, an exception was made which required four washes with SBB before washing with PBS and treating with elution buffer. The proteins were lyophilized before being analyzed by Western blotting using an anti-hnRNP M antibody.

### Proteomic analysis.

Digested peptides were subjected to LC-MS/MS in a custom-made 4-column LC system connected to an Orbitrap mass spectrometer (LTQ Orbitrap XL; Thermo Scientific, San Jose, CA) using previously described parameters (47). Tandem mass spectra were searched with the MS-GF+ database search tool (48) against bait (20 sequences) and *E. coli* K-12 (4,305 entries, downloaded on 18 June 2015) protein sequences in combination with human (68,485 entries, downloaded on 20 May 2015) or mouse (45,263 entries, downloaded on 20 May 2015) reference proteomes from the Uniprot Knowledge Base. Search parameters included tryptic digestion of at least one peptide terminus, methionine oxidation and cysteine alkylation (carbamidomethylation or addition of NEM, depending on the sample treatment) as variable modifications, and 20-ppm parent-mass tolerance. All peptides were filtered with MS-GF scores of ≤1.0E−8, and, to provide higher confidence, each protein further required ≥2 peptides and at least 1 peptide with an MS-GF score of ≤1.0E−10. Spectral counting was used for semiquantitation of putative interaction partner proteins, and high-confidence interactors were selected using the spectral count version of SAINTexpress (15).

### *Salmonella* infection of RAW 264.7 cells.

*Salmonella* Typhimurium (ATCC 14028) was used as the wild-type parental strain. A SrfH deletion mutant (Δ*srfH*) was constructed using the phage λRed recombination system (49). Cell culture infection was performed as previously described (50), with the following modifications: (i) macrophages were not pretreated with interferon gamma; and (ii) the bacterial inoculum was centrifuged on macrophage cells and washed with gentamicin (25 µg/ml), and time zero was recorded immediately after washing (i.e., without a further 30-min incubation).

### Transfections and coimmunoprecipitation.

HEK293T cells were transfected with plasmids encoding GFP alone or GFP-tagged WT SrfH (GFP::SrfH) and its truncated forms (amino acids 1 to 140 and 137 to 322) using X-fect transfection reagent (Clontech, Mountain View, CA). For pulldown assays, these transiently transfected HEK293T cells were lysed using 50 mM Tris-HCl (pH 7.5), 5 mM EDTA, 150 mM NaCl, 1% Triton X-100, and protease inhibitor. The postnuclear supernatant was generated by spinning the lysates at 13,000 rpm at 4°C. Meanwhile, anti-rabbit Dynabeads (Life Technologies) were conditioned by washing three times with PBS and incubation with rabbit polyclonal anti-GFP antibody (Santa Cruz Biotechnologies, Dallas, TX). The antibody was cross-linked to the beads, and the beads were incubated with the transfected HEK293T cell lysates overnight at 4°C. The next day, the beads were washed three times with lysis buffer and resuspended in sample buffer for SDS-PAGE analysis followed by Western blotting using either mouse monoclonal anti-GFP or anti-ERK2 antibodies (Cell Signaling Technologies, Danvers, MA).

### *In vitro* enzymatic assays.

A DeNEDDylase assay for analysis of SseL deubiquitinase activity was performed as described in detail previously (29). An *In vitro* cleavage assay was performed using purified SrfH and ERK2 (Novus Biologicals, Littleton, CO) mixed with 0.1% Triton X-100–PBS and incubation at 25°C with shaking for 4 h. Reaction was stopped with the addition of 4× LDS loading buffer with 5 mM TCEP with heating at 95°C for 10 min, and the reaction mixture was analyzed by Western blotting with anti-ERK2 antibody. Cleavage was assessed by quantification of lower-molecular-weight products along with the disappearance of the expected molecular-weight band in SrfH-treated samples. For *in vitro* deamidation assay, purified SrfH was incubated with ERK2, separated by SDS-PAGE, digested with endoproteinase Glu-C, and analyzed by LC-MS/MS, as described in detail in the Supplemental Material and Methods (see Text S1 in the supplemental material).

### Accession numbers.

All LC-MS/MS data files were deposited in the PRIDE archive-proteomics data repository (http://www.ebi.ac.uk/pride/archive/) under accession numbers PXD003943, PXD004002, and PXD003304.
