# Supplementary figures and images for: Identification of Novel Host Interactors of Effectors Secreted by Salmonella and Citrobacter
Source: mSystems. 2016 Jul 12;1(4):e00032-15. doi: 10.1128/mSystems.00032-15 (PMC5069955; doi:10.1128/mSystems.00032-15)

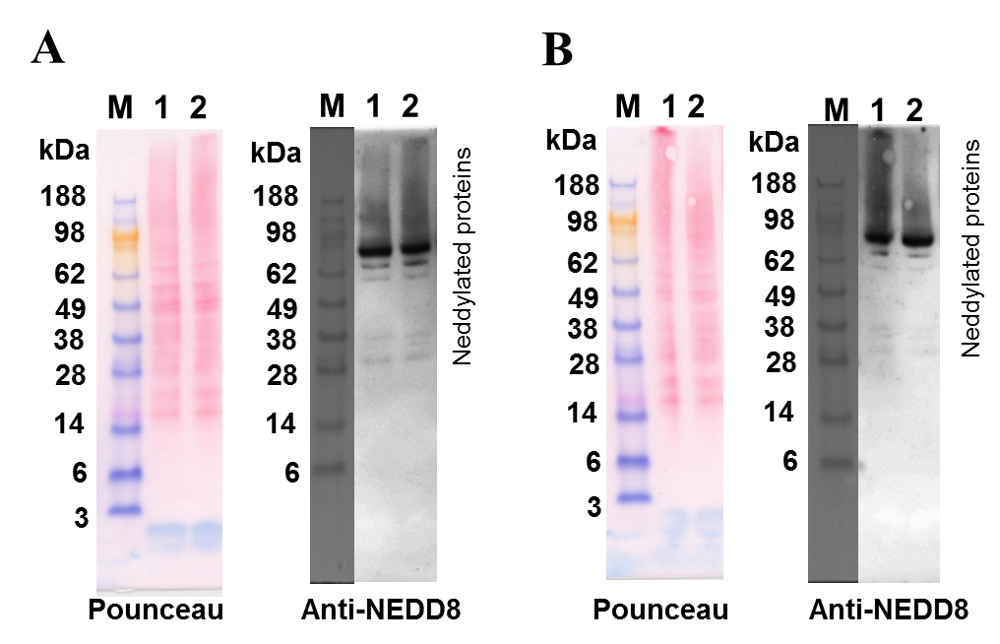

Supplement: Figure S1 [file sys004162037sf3.tif]

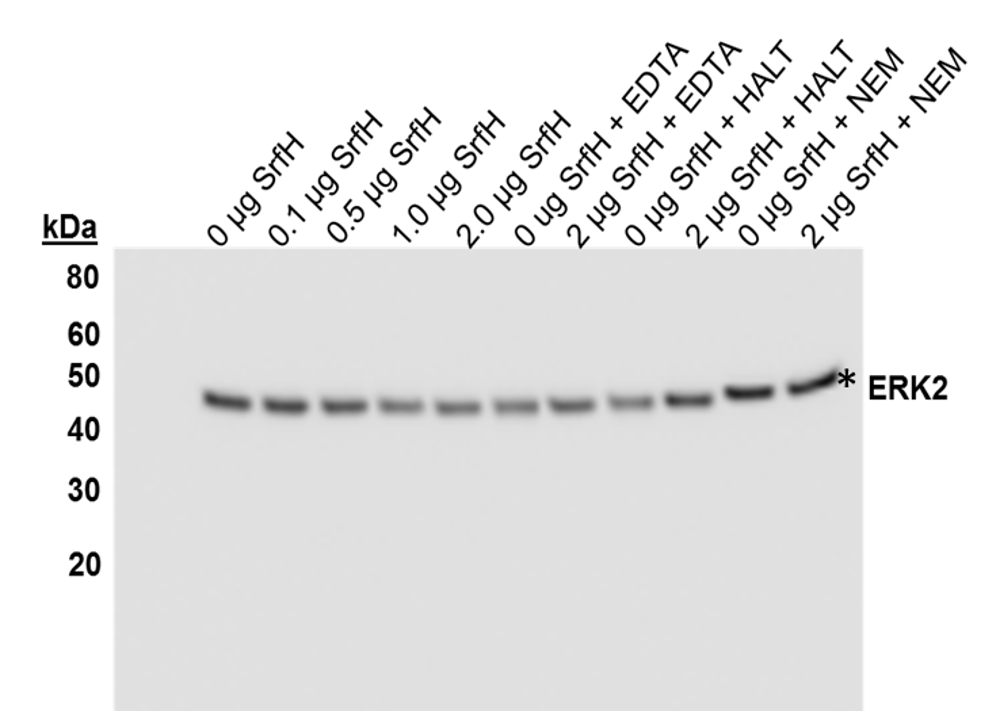

Supplement: Figure S2 [file sys004162037sf4.tif]

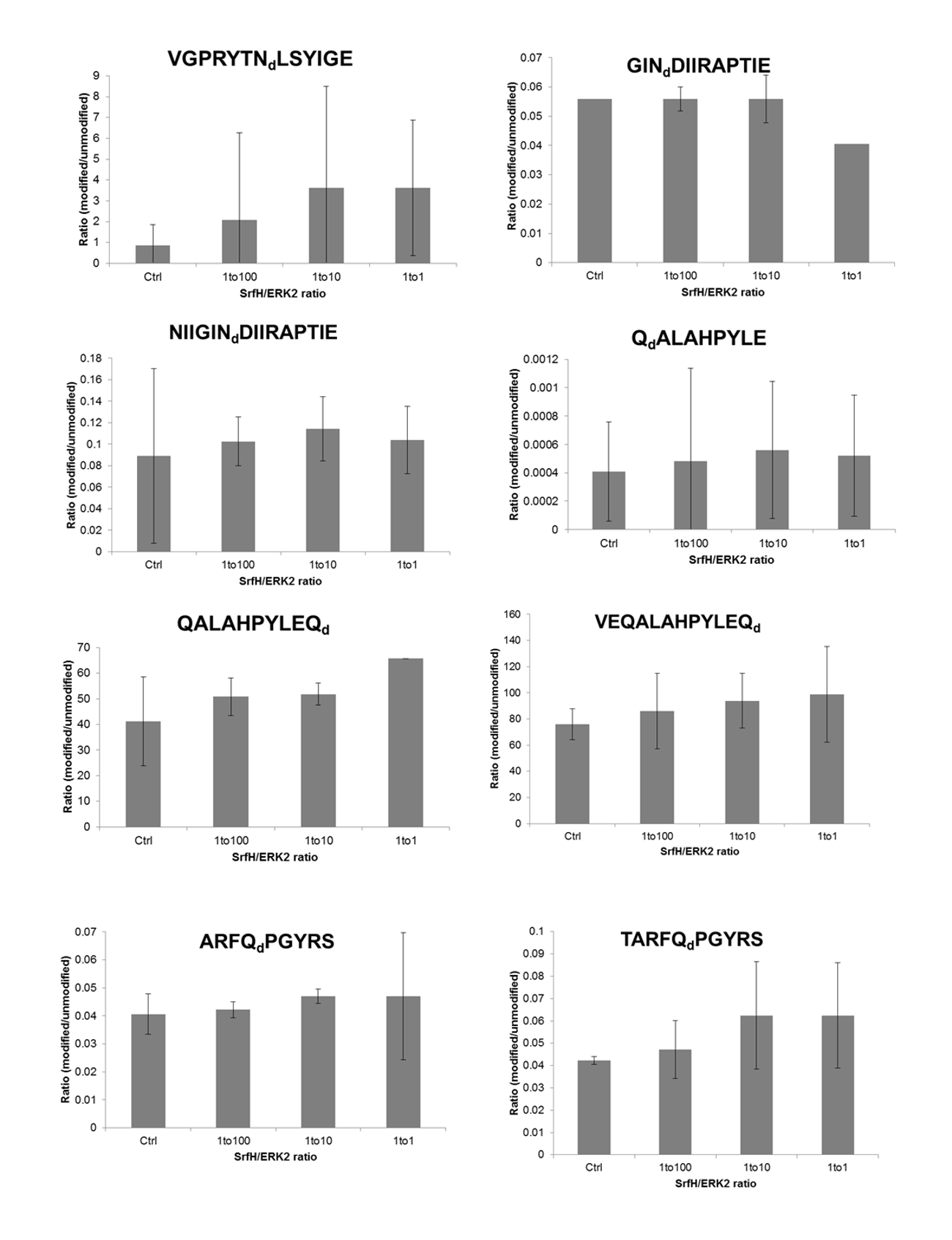

Supplement: Figure S3 [file sys004162037sf5.tif]

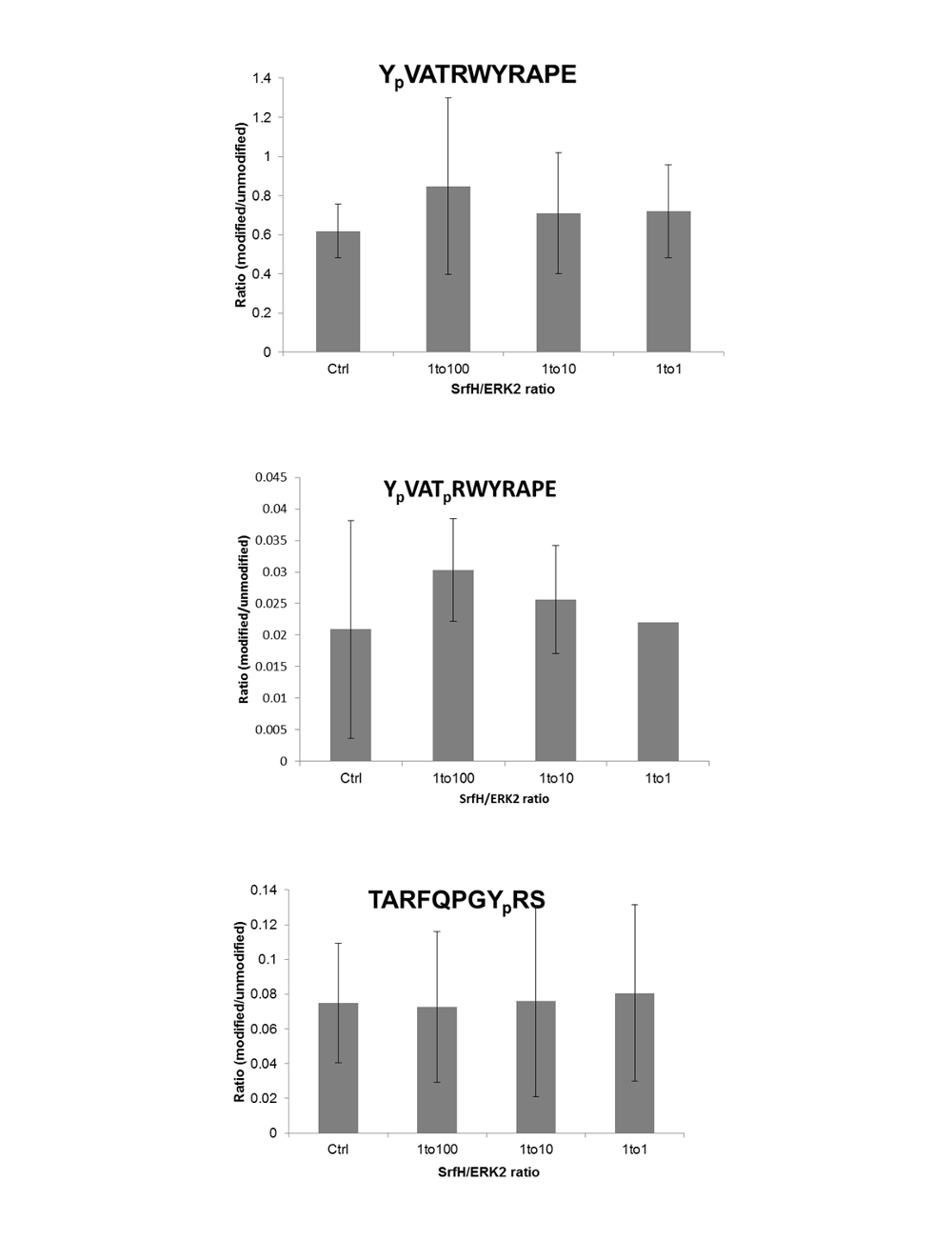

Supplement: Figure S4 [file sys004162037sf6.tif]
