# Supplementary material for: Identification of Novel Host Interactors of Effectors Secreted by Salmonella and Citrobacter
Source: mSystems. 2016 Jul 12;1(4):e00032-15. doi: 10.1128/mSystems.00032-15 (PMC5069955; doi:10.1128/mSystems.00032-15)
Supplement: Text S1 [file sys004162037s7.docx]

**Identification of novel host interactors of effectors secreted by *Salmonella* and *Citrobacter*: SrfH regulates host kinase ERK2 phosphorylation**

Ryan L Sontag^a¶^, Ernesto S Nakayasu^a¶^, Roslyn N Brown^a‖^, George S Niemann^b±^, Michael A Sydor^a^, Octavio Sanchez^a^, Charles Ansong^a^, Shao-Yeh Lu^c^, Hyungwon Choi^d^, Dylan Valleau^e^, Karl K Weitz^a^, Alexei Savchenko^e^, Eric D. Cambronne ^b^, and Joshua N Adkins^a^*

^a^Biological Sciences Division, Pacific Northwest National Laboratory, Richland, Washington, USA; ^b^Department of Molecular Microbiology & Immunology, Oregon Health & Science University, Portland, Oregon, USA; ^c^Department of Veterinary Microbiology & Pathology, Washington State University, Pullman, Washington, USA; ^d^Saw Swee Hock School of Public Health, National University of Singapore, Singapore. ^e^Department of Chemical Engineering and Applied Chemistry, Banting and Best Department of Medical Research, Midwest Centre for Structural Genomics, University of Toronto, Toronto, Ontario M5G 1L6, Canada.

^¶^These authors contributed equally to this work.

Current address: ^‖^ Cepheid, Inc., 904 E Caribbean Dr., Sunnyvale, CA 94089, USA. ^±^ Calypte Biomedical, Portland, Oregon, USA.

*Address correspondence Joshua N. Adkins, Joshua.Adkins@pnnl.gov

**Supplemental Material and Methods**

***In vitro* deamidation and dephosphorylation by SrfH**

Purified ERK2 (Novus Biologicals, Littleton, CO) and SrfH (produced in-house) were combined (ERK2:SrfH, m:m ratio) at 1:1, 1:10, and 1:100 in 20 mM Tris-HCl, 10mM MgCl_2_, and 1 mM EDTA. The mixture was incubated at 37 °C with shaking overnight. The reaction was stopped by addition of 4x lithium dodecylsulfate (LDS) sample loading buffer and 5 mM tris(2-carboxyethyl)phosphine (TCEP) before heating at 95 °C for 10 minutes. After cooling, the samples were separated on a 4-12% bis-tris polyacrylamide gel, and stained with GelCode Blue (Pierce). Bands corresponding to the molecular weight of ERK2 were excised and cut into pieces < 1 mm^3^. Gel pieces were destained with 50% acetonitrile and 50%-50 mM NH_4_HCO_3_ until clear and then fully dehydrated with 100% acetonitrile. Gel pieces were dried in a SpeedVac, before rehydrating with endoproteinase GluC (Staphylococcus aureus Protease V8) diluted 1:10 (m/m) the buffer provided by the manufacturer (New England BioLabs, Ipswith, MA) and incubated overnight at 37 °C with shaking. The peptides were recovered by extracting the gel pieces 3x with 5% formic acid / 50% ACN for 20 minutes at room temperature. The solution was then dried to completeness in a SpeedVac and resuspended in 0.1% formic acid for LC-MS/MS analysis as described above.

LC-MS/MS runs were processed with MaxQuant v1.5.2.8 (1). Peptide identification was performed by searching tandem mass spectra against the *E. coli* K12 appended to the recombinant ERK2 sequence. Search parameters included semispecific endoproteinase GluC with missed cleaved sites allowed, and the following variable modifications: protein N-terminal acetylation, deamidation of glutamine and asparagine, methionine oxidation and phosphorylation on serine, threonine and tyrosine. The mass tolerance for peptide identification and feature detection were set as default by the software. For quantification, identified peptides were aligned and matched between runs with 3 min matching window and 20 min alignment window. The extracted peak areas for each modified peptide were normalized by dividing by the area of the respective unmodified counterpart. Significant changes were determined by Student T-test on Excel assuming equal variance (Microsoft, Redmond, WA).

**Supplemental Results and Discussion**

**Potential activities of SrfH on ERK2**

The recent elucidation of SrfH tertiary structure revealed a putative active site that has homology to variety of possible functions including a cysteine protease, glutamine deamidase, transglutaminase, N-acetyltransferase or glycanase (2). In addition, Mc Laughlin *et al*. also noticed that cysteine (C178) in the putative active site was required for preventing dissemination into the host spleen but not for binding to IQGAP1 (3), suggesting that this residue might be important for enzymatic activity. Bhaskaran and Stebbins used generic substrates in an attempt to determine the catalytic activity of SrfH, but were unable to confirm enzymatic function; raising the possibility SrfH would only target specific substrates (2). To test the possibility of SrfH being a cysteine protease that cleaves ERK2, we incubated purified, recombinant ERK2 with SrfH and searched for cleavage products by western blot. Parallel reactions with the addition of EDTA (metalloproteinase inhibitor), NEM (cysteine protease inhibitor) and protease inhibitor cocktail were performed as controls. We were unable to detect any cleavage of ERK2 by SrfH **(Figure S2A)**. We also looked at the possibility that SrfH could be a glutamine deamidase, thus we incubated recombinant ERK2 with SrfH, separated the products by SDS-PAGE, digested with endoproteinase Glu-C and analyzed the peptides by LC-MS/MS. We were able to identify and quantify eight unique deamidated peptides, corresponding to five deamidation sites on glutamine and asparagine residues. However, none of the identified deamidated peptides were augmented by treating with SrfH (**Figure S3**). Despite our inability to demonstrate an activity, we cannot dismiss the possibility that specific post-translational modifications like S-palmitoylation (4) or other cofactors that are lacking in an *in vitro* assay might be required or that SrfH has a completely different activity.

**References**

1. **Cox J, Mann M.** 2008. MaxQuant enables high peptide identification rates, individualized p.p.b.-range mass accuracies and proteome-wide protein quantification. Nat Biotechnol **26:**1367-1372.

2. **Bhaskaran SS, Stebbins CE.** 2012. Structure of the catalytic domain of the Salmonella virulence factor SseI. Acta Crystallogr D Biol Crystallogr **68:**1613-1621.

3. **McLaughlin LM, Govoni GR, Gerke C, Gopinath S, Peng K, Laidlaw G, Chien YH, Jeong HW, Li Z, Brown MD, Sacks DB, Monack D.** 2009. The Salmonella SPI2 effector SseI mediates long-term systemic infection by modulating host cell migration. PLoS Pathog **5:**e1000671.

4. **Hicks SW, Charron G, Hang HC, Galan JE.** 2011. Subcellular targeting of Salmonella virulence proteins by host-mediated S-palmitoylation. Cell Host Microbe **10:**9-20.
